# Exploring heterogeneity in PTSD symptoms and associated predictors and outcomes in Afghanistan veterans: A latent profile analysis

**DOI:** 10.1080/08995605.2024.2345580

**Published:** 2024-05-06

**Authors:** Line Rønning, Frederick Anyan, Odin Hjemdal, Hans Jakob Bøe, Andreas Espetvedt Nordstrand, Holly B. Herberman Mash, James A. Naifeh

**Affiliations:** a Department of Psychology, Norwegian University of Science and Technology, Trondheim, Norway; b Institute of Military Psychiatry, Norwegian Armed Forces Joint Medical Services, Oslo, Norway; c Department of Psychology, University of Oslo, Oslo, Norway; d Center for the Study of Traumatic Stress, Department of Psychiatry, Uniformed Services University of the Health Sciences, Bethesda, Maryland, USA; e Henry M. Jackson Foundation for the Advancement of Military Medicine, Inc., Bethesda, Maryland, USA

**Keywords:** Military, PTSD, social support, suicide risk, healthcare

## Abstract

Research on posttraumatic stress symptoms (PTSS) typically focuses on diagnosis or symptom severity, however, this overlooks the variety of symptom patterns that exist. Latent profile analysis was used to explore PTSS profiles in a sample of Norwegian Afghanistan veterans (*n* = 4052, 91.7% males). Multinomial logistic regression analyses were conducted to examine predictors and outcomes associated with PTSS profile membership. Three profiles emerged: *Low Symptoms* profile (85%); *High Numbing and Arousal* profile (13%); and *High Symptoms* profile (2%). Being female, lower number of deployments, barriers to disclose war-related experiences, and higher number of potentially morally injurious events (PMIEs) were associated with belonging to the *High Symptoms* profile compared to the *High Numbing and Arousal* (Male gender: OR = 0.37, *p* < .05; Number of deployments: OR = 0.68, *p* < .05; Barriers to disclose: OR = 1.39, *p* < .001; PMIEs: OR = 1.15. *p* < .05), or *Low Symptoms* profile (Male gender: OR = 0.36, *p* < .05; Number of deployments: OR = 0.67, *p* < .01; Barriers to disclose: OR = 1.80, *p* < .001; PMIEs: OR = 1.32. *p* < .001). Participants in the *High Symptoms* profile had the highest probability of mental health service use (0.37) and endorsing suicidal ideation (0.38), compared to the two other profiles (*p* < .01). Participants in the *High Numbing and Arousal* profile had a higher probability of seeking professional mental health care (0.17), endorsing suicidal ideation (0.16), and reporting more suicide attempts compared to the *Low Symptom* profile (0.02 vs. 0.00, *p* < .001). These findings highlight the importance of considering the heterogeneity of PTSS profiles and understanding the predictors and responses of individuals who exhibit elevated PTSS symptoms.

**What is the public significance of this article?—**This study emphasizes the significance of considering the heterogeneity in PTSD symptom presentation during the assessment, diagnosis, and treatment of veterans and military personnel. This underscores the need for a more nuanced and tailored approach to care, as veterans may fall into different symptom profiles, each requiring unique interventions. Specifically, veterans and military personnel who do not meet the standard diagnostic criteria for PTSD might still suffer from distinct symptom elevations, have heightened suicide risk, and may need assistance and help from mental health care professionals.

Individuals with posttraumatic stress disorder (PTSD) can have remarkably different symptom presentations (Galatzer-Levy & Bryant, 2013; Murphy et al., 2019), and such heterogeneity is likely to affect treatment outcomes (e.g., Currier et al., 2014; Murphy & Smith, 2018; Phelps et al., 2018). This is particularly pertinent for military personnel and veterans with PTSD who appear to respond less favorably to treatment compared to other traumatized populations (Bradley et al., 2005; Steenkamp et al., 2015). Hence, understanding the heterogeneity of PTSD symptom profiles or subtypes can be crucial for developing targeted interventions for trauma-exposed veterans and military personnel.

Most research on PTSD has relied on psychometric measures to determine the presence or absence of a PTSD diagnosis, or to examine the severity of PTSD symptoms as a composite score to measure the burden of PTSD symptoms. While such variable-centered approaches can provide insight into the prevalence or severity of PTSD in a population, they do not allow for a deeper understanding of the ways in which PTSD symptoms may vary within that population (Murphy et al., 2019). This has led researchers (e.g., Campbell et al., 2020; Contractor et al., 2017, 2020; Hebenstreit et al., 2014; Jongedijk et al., 2019; Murphy et al., 2019) to use person-centered approaches, such as latent class analysis (LCA; assessing binary indicators) or latent profile analysis (LPA; assessing continuous indicators), to identify groups of individuals who share particular traits. The current study favored LPA due to its use of continuous indicators, which allows for a more nuanced assessment of symptom severity and dimensionality, not only symptom presence or absence.

While several LPA studies have used all available posttraumatic stress symptom items to investigate PTSD in trauma-exposed samples (i.e., Hebenstreit et al., 2015; Nugent et al., 2019; Zhou et al., 2019), previous research on military personnel and veterans have incorporated items measuring depressive symptoms and general anxiety symptoms alongside the PTSD indicators (i.e., Armour et al., 2015; Contractor et al., 2015). It is also important to note that these military and veteran samples are predominantly from North America (Canada and U.S.), which may not fully apply to another military contexts, such as the Norwegian Armed Forces. This geographical bias in research leaves a significant gap in the understanding of PTSD symptoms in military personnel and veterans from countries outside North America, particularly related to continuous PTSD indicators and their associations with risk and protective factors. To address this gap, the current study focused on a sample of Norwegian Afghanistan veterans to contribute to a more comprehensive and global understanding of PTSD in a military context.

One robust risk factor for PTSD among veterans is war zone trauma exposure, with specific focus on the qualitative aspects of these exposures, as different types of combat-related traumatic events may increase risk for different types of posttraumatic stress symptoms (Shea et al., 2017). For instance, exposure to personal life threat was found to be associated with hyperarousal symptoms, whereas exposure to death or severe injury of others predicted depression symptoms (Shea et al., 2017). In addition to the characteristics of war zone trauma, social support plays a critical role in the development and maintenance of PTSD (e.g., Nordstrand et al., 2020; Zalta et al., 2021). Specifically, higher levels of social support have been associated with lower PTSD symptom severity (Zalta et al., 2021), and personal barriers to share war zone experiences were related to higher subsequent posttraumatic stress reactions and psychological distress (Nordstrand et al., 2020; Thoresen et al., 2014).

It is well-established that PTSD increases the risk of suicidal ideation and attempt (Brown et al., 2016; Marshall et al., 2001). This association has been found across various populations, including civilians, veterans, and active-duty military (Brown et al., 2016, 2018; Panagioti et al., 2009). Such understanding and knowledge are necessary to aid clinicians in identifying patients who may be at higher risk for suicide, as military personnel and veterans are at an increased risk for suicide compared with the general population, with rates of suicide among this at-risk population steadily increasing since the beginning of the wars in Afghanistan and Iraq (Kang et al., 2015; Schoenbaum et al., 2014). However, military personnel with mental health problems may experience barriers to receiving mental health services (Hoge et al., 2004). These barriers can hinder both the initiation and continuation of mental health treatment (Naifeh et al., 2016). Research indicates that many soldiers with mental disorders do not believe they require treatment, and among those who do recognize the need for help, they often face multiple attitudinal barriers (e.g., fear of stigma, perceived ineffectiveness of treatment, desire to handle problems on one’s own), as well as structural barriers (e.g., financial constraints or inconvenience) which prevent them from seeking help (Naifeh et al., 2016). As such, knowledge about those who seek help from mental health care, and those who do not, is necessary to identify and develop effective, targeted interventions and improve outreach strategies.

The aim of this study was three-fold. First, using LPA, we aimed to identify profiles of PTSD symptoms in a large sample of Norwegian Afghanistan veterans using the 17 items of the PTSD Checklist for DSM-IV, Military Version (PCL-M; Weathers et al., 1993). Second, we examined how the different symptom profiles were associated with predictors not previously investigated in LPA studies, including qualitative aspects of war zone trauma exposure, social support and network size, and barriers to disclose traumatic experiences. Third, we determined which symptom classes best represent the subset of veterans who report suicidal ideation, suicide attempt, and those who seek mental health care.

Consistent with previous research exploring different PTSD symptom presentations in veteran and military populations, we hypothesized finding three (Armour et al., 2015; Contractor et al., 2015; Steenkamp et al., 2012) or four (Campbell et al., 2020; Contractor et al., 2020; Hebenstreit et al., 2014; Maguen et al., 2013) latent profiles of PTSD symptoms. We also hypothesized that lack of social support and barriers to disclose traumatic experiences from Afghanistan, in addition to the qualitative aspects of war zone trauma exposure, would uniquely contribute to the emergence of these profiles, such that those characterized by higher symptom severity would report less social support, higher barriers to disclose, and would experience a greater impact of war zone trauma. Finally, we hypothesized that the profiles would substantially differ for those who reported suicide ideation, suicide attempts, and sought mental health care, such that those in the higher symptom severity profiles would report greater suicide risk and less mental health service use.

## Material and methods

### Participants and procedure

All Norwegian military personnel who had been deployed to Afghanistan between late 2001 and the end of 2011 were invited to participate. This included both currently serving military personnel and former military who had been deployed to Afghanistan. A total of 7155 personnel of both genders were identified to fit the requirement by the Recruiting Department of the Norwegian Armed Forces. The identified personnel received written invitations to complete a 20-page questionnaire, either on paper, by mail, or on a webpage. An incentive to respond was participation in a lottery of three sport watches. The data collection phase lasted thirteen weeks, from the 20th of February to the 24th of May 2012.

Of the invited personnel, 4225 (59%) responded in total: 1931 (46%) by mail and 2294 (54%) on the web. Of the responses, 173 (4.1%) were either incomplete or active refusals. The non-responders and those with incomplete responses and active refusals totaled 3103 (43.4%) persons. In all, 4052 individuals returned fully completed questionnaires, resulting in a final response rate of 56.6%.

All procedures, storing, distribution, and collection of data were made in accordance with the legislation regulating the Norwegian Armed Forces Health Registry. The study was approved by the Regional Committee for Medicine and Health Research Ethics of South-East Norway.

### Measures

#### PTSD symptoms

Veterans completed the PTSD Checklist for DSM-IV, Military Version (PCL-M; Weathers et al., 1993), which is a 17-item self-report measure assessing symptoms of military-related PTSD. The symptoms are categorized into three clusters: symptoms of intrusive recollections, avoidant/numbing symptoms, and hyperarousal symptoms (American Psychiatric Association, 1994; Weathers et al., 1993). Participants rated the severity of their PTSD symptoms over the past month on a five-point Likert-type scale (0 = *Not at all* through 4 = *Extremely*). For the initial analysis, a value equal to or greater than 44 indicated probable PTSD diagnosis (e.g., Blanchard et al., 1996). However, research has demonstrated that lower cutoff values than 44 may offer better diagnostic efficiency, especially when the PCL is used in primary care settings (Bliese et al., 2008). Consequently, this study also investigated a cutoff of 30. The PCL-M has demonstrated good psychometric properties in veteran samples (Wilkins et al., 2011). In the current study, internal reliability was good (*α* = 0.89).

#### Suicide ideation and suicide attempt

Post-deployment suicide ideation and lifetime suicide attempt were assessed with the following questions: “After returning from Afghanistan, have you been depressed/down in such a way that you have had thoughts of taking your own life?” and “Have you ever attempted suicide?.” Each item was scored dichotomously (1 = yes, 0 = no).

#### Health care for mental health problems

The veterans were also asked about their use of mental health services by the question, “Have you ever sought health care for mental health problems?.” The answer options were dichotomous, indicating whether the veterans had sought help (1 = yes, 0 = no).

#### War zone traumatic exposure

War zone traumatic experiences were measured by 12 items covering three stressor types that military personnel might experience when deployed in war zones: personal threat, moral challenges, and witnessing, which respectively related to items covering life-threatening situations, experiences that transgressed moral beliefs, and the witnessing of major suffering. Each of the 12 items had a 5-point Likert response format, with response options of 0 – “*not experienced*;” 1 – “*experienced 1–2 times*;” 2 – “*experienced 3–12 times*;” 3 – “*experienced 13–50 times*;” and 4 – “*experienced 50*+ *times*.” Item selection was based on previous studies utilizing the same sample (Nordstrand et al., 2019). The three war zone trauma stressors were labeled Personal Threat (4 items; *n* = 4015, *M* = 5.31, *SD* = 1.87), Potentially Morally Injurious Events (PMIEs) (3 items; *n* = 4012, *M* = 3.98, *SD* = 1.43), and Traumatic Witnessing (5 items; *n* = 4015, *M* = 7.59, *SD* = 2.39), and mirror the war zone stressor items utilized in a previous study (Nordstrand et al., 2019).

#### Social connections and strains

To assess perceived social strains during and following deployment, several variables were used. The measure of ***social support*** was adapted from the Oslo Social Support Scale 3 (Dalgard, 1996; Meltzer, 2003). This inventory captures perceptions of social support, including structural support (size of network), perceived support (perceived availability and the satisfaction with support), and enacted support (actions taken to receive aid/support). Minor changes in the wording of some items were required to adapt them to the post-deployment context of the veterans: “How many people are you so close to that you can count on them if you have great personal problems?;” “People show interest in what I have experienced in Afghanistan;” “In the time since returning home, I have had someone I can lean on if I run into problems.” The participants rated the first item on a 4-point Likert response format with the following response options: 1 – *“none”*; 2 – *“one to two”*; 3 – *“three to five”*; 4 – *“six or more”*. The latter two items were rated with a 5-point Likert response format with these response options: 1 – *“Completely Disagree”*; 2 – *“Disagree Somewhat”*; 3 – *“Neither (neutral)”*; 4 – *“Agree Somewhat”*; 5 – *“Completely Agree.”* The total sum score of the three questions ranged from 3 to 14 (*n* = 4028, *M* = 10.24, *SD* = 1.43, *α* = .51), with high values representing strong levels of social support and low values representing weak levels.

***Barriers to disclose*** traumatic experiences from Afghanistan were assessed with the following three items: “I experienced incidents in Afghanistan which I have not been able to tell others about, even those closest to me;” “I have/had problems that I am not able to share with family or friends;” and “There is no one at home who is able to understand what I have experienced.” Each of the three items had a 5-point Likert response format with the following response options: 1 – *“Completely Disagree”*; 2 – *“Disagree Somewhat”*; 3 – *“Neither (neutral)”*; 4 – *“Agree Somewhat”*; 5 – *“Completely Agree”*. The sum score ranged from 3 to 15 (*n* = 4024, *M* = 6.96, *SD* = 2.73), with higher scores indicating greater barriers to disclose.

Furthermore, ***cohabitation status*** was assessed using the following response options: single, cohabitation, married/partner, divorced/separated, widow/widower, and other.

Finally, information regarding number of deployments, gender, and age was obtained through the Norwegian Armed Forces Health Registry, which collects such data on all individuals who serve in the Norwegian Armed Forces.

### Statistical analyses

Latent Profile Analysis (LPA) with continuous indicators of symptoms of military-related PTSD was performed in Mplus 8.8 (Muthén & Muthén, 1998–2022). The 17 items of the PCL-M were used as indicators in the analysis. The utilization of all PTSD items available for the LPA is in line with previous studies on both military personnel and veteran samples (Armour et al., 2015; Contractor et al., 2015) and other trauma exposed samples (Contractor et al., 2017; Hebenstreit et al., 2015; Nugent et al., 2019; Zhou et al., 2019). To make use of all available data, full-information maximum likelihood was used with robust estimation (MLR) due to non-normality. Progressively larger numbers of latent profile solutions were explored to determine the optimal solution. To avoid convergence on local maxima solutions, the models were estimated using 1,000 random sets of start values with 100 iterations and the 200 best solutions retained for final stage optimization. A variety of model fit statistics, substantive meaningfulness of the profiles, and their theoretical interpretability were analyzed to determine the optimal solution. We examined fit statistics with classification accuracy so that the average probability of belonging to the most likely profile should be high, and the average probability of belonging to the other profiles should be low. Model fit statistics included the Akaike information criterion (AIC), Bayesian Information Criterion (BIC), sample-size adjusted BIC (ABIC), Vuong-Lo-Mendell-Rubin Likelihood Ration test (LMR-LRT), Lo-Mendell-Rubin Adjusted Likelihood Ratio test (ALMR-LRT), and the Bootstrapped Likelihood Ratio test (BLRT). The AIC, BIC and ABIC provide relative improvement in fit information when comparing models. The LMR-LRT, ALMR-LRT and BLRT compare different likelihood ratio tests that quantify the comparisons between the current model solution to a model solution with one fewer class. The BLRT outperforms LMR-LRT and the other information criteria statistics in most cases (Nylund et al., 2007). We sought a model with lower values for all criterion indices, but higher entropy values. Model fit indices in combination with substantive meaningfulness of profiles, and theoretical interpretability guided the final model selection (Grimm et al., 2016; Wickrama et al., 2021).

Once the optimal unconditional model solution was determined, relevant predictors (using the R3STEP procedure in Mplus) and outcome variables (using the Bolck – Croon – Hagenaars, BCH procedure in Mplus) were simultaneously included in multivariable models to determine which variables distinguish between profile differences and how profile membership predict relevant outcomes. The R3STEP and BCH result in less biased parameter estimates while maintaining a stable unconditional model solution and interpretable coefficients for the covariates (Asparouhov & Muthén, 2014). The BCH uses Wald tests to compare the mean levels of outcome variables across profiles (Bakk & Vermunt, 2016).

## Results

### Descriptive statistics

In total, 4052 participants (91.66% males) completed the questionnaire. Their ages ranged from 21–72 years, with a mean age of 35.42 years (*SD* = 9.29). The majority of the participants reported being married or in a relationship (70.25% in a relationship/married versus 29.75% reported being single, divorced/separated, widowed, or other). The highest attained education level was university for 49.28%, high school for 35.87%, vocational training for 12.67%, and elementary school for 2.18%. The mean number of Afghanistan deployments was 1.76 (*SD* = 1.69). The scores of PCL-M ranged from 17–79, with a mean score of 20.95 (*n* = 4007, *SD* = 6.09). A total of 48 participants (1.20%) scored equal to or greater than 44, indicating probable PTSD diagnosis, whereas 319 participants (7.96%) scored equal to or greater than 30. A total of 164 participants (4.07%) had experienced suicidal ideation after deployment, and 26 (0.65%) reported a lifetime suicide attempt. Finally, a total of 243 (6.04%) had requested professional healthcare for mental health problems.

### Identification of latent profiles

The model with the 1-profile solution showed the largest AIC, BIC, and ABIC values, indicating least favorable fit. The LMR LR test, ALMR LR test, and BLRT in the 2-profile solution all had *p*-values < .05, suggesting rejecting a single-profile solution in favor of at least two profiles. Next, we considered the 3- and 4-profile solutions, with both showing non-significant LMR LR and ALMR LR tests, but significant BLRT, as was the case for all competing profile solutions. Relative fit indices in the 4-profile solution were substantially lower than in the 3-profile solution. However, one profile in the 3-profile solution and two profiles in the 4-profile solution emerged that contained a relatively lower proportion of the sample (≤3% of the total sample). When considering the 5- and 6-profile solutions, profile distinctiveness was unclear and the substantive interpretations of the profiles were not of much theoretical interest (e.g., no qualitatively different profiles emerged). Additionally, the 6-profile solution contained several profiles with a relatively lower proportion, making it unfavorable compared to the 5-profile solution.

Consequently, we returned to the 3- and 4-profile solutions and compared them to the 5-profile solution. Classification accuracy was wider in the 5-profile solution than the 4-profile solution; hence, we limited our selection to the 3- and 4-profile solutions, both having very high Entropy values (>.90). Overall, the average probability of belonging to the most likely profile was narrower in the 4-profile solution (.995–1.000) than in the 3-profile solution (.969–.993), suggesting that there was high classification accuracy in the 4-profile solution as well as high average probability of belonging to the most likely profile, and low average probability of belonging to other profiles in the 4-profile solution than in the 3-profile solution.

We tested validity evidence using the R3STEP and BCH approach with several covariates, including age, gender, number of military deployments, cohabitation status, social support, barriers to disclose traumatic experiences, and war zone traumatic experiences for the derived profiles in the 3- and 4-profile solutions. We found that two of the profiles for the 4-profile solution (3% and 11%) were not distinctively different from each other, and thus, could constitute one profile, whereas derived profiles in the 3-profile solution not only showed quantitative differences, but also qualitative differences. Thus, guided by theoretical interpretability and substantive meaningfulness of profile plots, we favored the 3-profile solution, which showed a reasonable representation of the data and was more parsimonious Table 1.

**Table 1.** Model fit indices for latent profile analysis of PTSD symptoms among Norwegian military veterans.

	AIC	BIC	ABIC	Entropy	LMR LR Test p-values	ALMR LR Test p-value	Sample proportion per class	Classification accuracy	BLRT p-value
1-Profile	105703.099	105917.156	105809.119				4007		
2-Profiles	88781.589	89108.970	88943.738	.983	.0061	.0064	(3617; 90%), (390; 10%)	.980–.997	.000
3-Profiles	81928.969	82369.675	82147.246	.977	.2039	.2053	(3392; 85%), (522; 13%), (93; 2%)	.969–.993	.000
4-Profiles	73028.749	73582.779	73303.155	.986	.7521	.7522	(114; 3%), (457; 11%), (3409; 85%), (27; 1%)	.995–1.000	.000
5-Profiles	70021.943	70689.298	70352.477	.985	.7687	.7687	(150; 4%), (113; 3%), (3345; 83%), (372; 9%), (27; 1%)	.961–1.000	.000
6-Profiles	66069.082	66849.761	66455.744	.982	.4483	.4483	(35; 1%), (105; 3%), (3216; 80%), (439; 11%), (193; 4%), (19; 1%)	.944–1.000	.000

AIC = Akaike information criterion; BIC = Bayesian information criterion; ABIC = Sample-size adjusted BIC; LMR LR = Vuong-Lo-Mendell-Rubin Likelihood Ratio Test; ALMR LR = Lo-Mendell-Rubin Adjusted LRT Test; BLRT = Bootstrap likelihood ratio test.

### Interpretation of latent profiles

**Figure 1.**
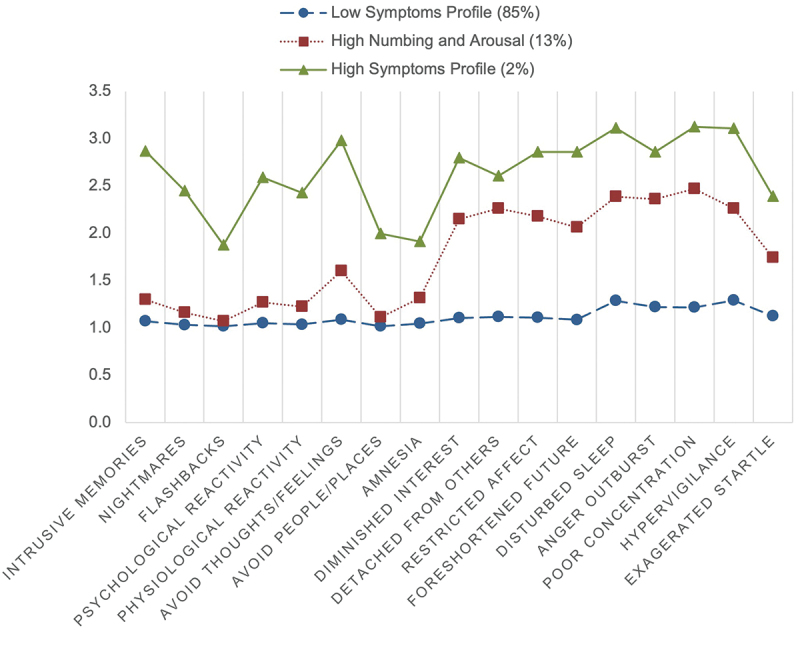
Profile plot showing levels of PTSD symptoms from the PCL-17.

**Figure 2.**
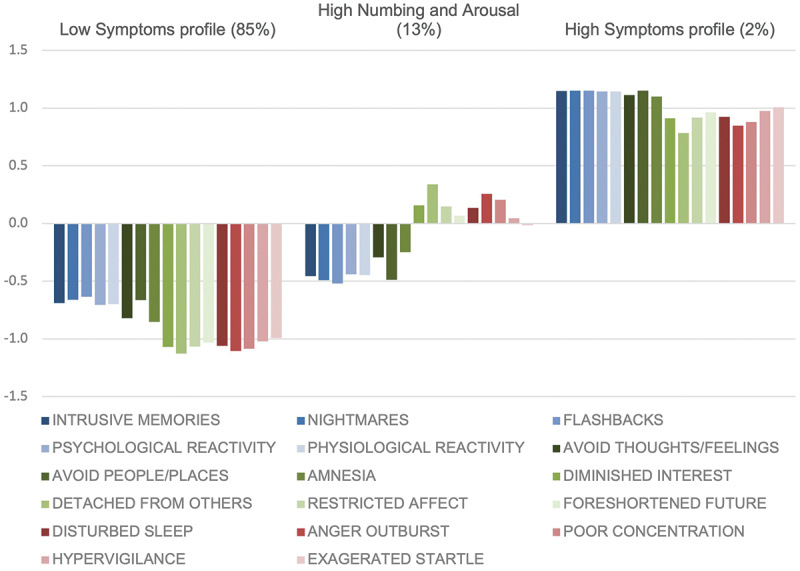
Bar graph showing standardized profile scores of PTSD symptoms from the PCL-17.

Participants exhibiting Profile 1 (85%) reported consistently lower levels of all 17 symptom indicators. As individuals identified by this profile reported consistently lower PTSD symptom levels, this category was designated as the *Low symptoms* profile. Profile 2 (13%) included low symptom levels for the domains of reexperiencing and avoidance, but high symptom levels for the domains of numbing and arousal. This profile was therefore designated as the *High Numbing and Arousal* profile. Profile 3 (2%) included higher levels of all 17 symptom indicators and was thus designated as the *High Symptoms* profile. Figure 1 displays a graphical representation of the profiles, and Figure 2 shows the standardized profile scores.

### Predictors of latent profile membership

Results from multinomial logistic regression analyses examining between-profile differences with relevant predictors are displayed in Table 2. The reference profile was switched across regressions so that all pairwise comparisons were made. Gender (Males) and higher number of deployments were associated with lower odds of belonging to the *High Symptoms* profile compared to the *Low Symptoms* (odds ratio [OR] = 0.36, 95% CI [0.153, 0.971] for gender; OR = 0.67, 95% CI [0.500, 0.894] for number of deployments), or *High Numbing and Arousal* profile (OR = 0.37, 95% CI [0.142, 0.985] for gender; OR = 0.68, 95% CI [0.507, 0.911] for number of deployments).

**Table 2.** Multinomial logistic regression parameters predicting profile membership.

	High Numbing and Arousal profile		High Symptom profile	
Predictors	OR	95% CI	OR	95% CI
Reference Class = Low Symptoms profile				
Gender (Males)	1.03	[0.662, 1.606]	0.36*	[0.153, 0.971]
Age	0.98*	[0.965, 0.991]	0.97	[0.938, 1.006]
Number of deployments	0.98	[0.924, 1.048]	0.67**	[0.500, 0.894]
Cohabitation	0.84	[0.666, 1.069]	0.58*	[0.355, 0.955]
Social support	0.78***	[0.735, 0.824]	0.73***	[0.656, 0.821]
Barriers to disclose	1.30***	[1.234, 1.360]	1.80***	[1.591, 2.041]
Personal threat	1.06	[0.997, 1.122]	1.05	[0.924, 1.118]
Traumatic witnessing	1.03	[0.981, 1.086]	1.09	[0.986, 1.208]
Potentially morally injurious events	1.15***	[1.070, 1.238]	1.32***	[1.158, 1.511]
Reference Class = High Numbing and Arousal profile				
Gender (Males)			0.37*	[0.142, 0.985]
Age			0.99	[0.959, 1.030]
Number of deployments			0.68*	[0.507, 0.911]
Cohabitation			0.69	[0.417, 1.144]
Social support			0.94	[0.842, 1.056]
Barriers to disclose			1.39***	[1.230, 1.573]
Personal threat			0.99	[0.872, 1.126]
Traumatic witnessing			1.06	[0.952, 1.174]
Potentially morally injurious events			1.15*	[1.014, 1.302]

*p < .05; **p < .01; ***p < .001.

Additionally, cohabitation and social support were associated with lower odds of belonging to the *High Symptoms* profile than the *Low Symptoms* profile (OR = 0.58, 95% CI [0.355, 0.955] for cohabitation; OR = 0.73, 95% CI [0.656, 0.821] for social support), whereas barriers to disclose and experiencing PMIEs were associated with higher odds of belonging to the *High Symptoms* profile than the *Low Symptoms* profile (OR = 1.80, 95% CI [1.591, 2.041] for barriers to disclose; OR = 1.32, 95% CI [1.158, 1.511] for PMIEs) or *High Numbing and Arousal* profile (OR = 1.39, 95% CI [1.230, 1.573] for barriers to disclose; OR = 1.15, 95% CI [1.014, 1.302] for PMIEs).

Further, barriers to disclose, and PMIEs were associated with greater odds of belonging to the *High Numbing and Arousal* profile than the *Low Symptoms* profile (OR = 1.30, 95% CI [1.234, 1.360] for barriers to disclose; OR = 1.15, 95% CI [1.070, 1.238] for PMIEs). Older age and more social support were associated with lower odds of belonging to the *High Numbing and Arousal* profile than the *Low Symptoms* profile (OR = 0.98, 95% CI [0.965, 0.991] for age; OR = 0.78, 95% CI [0.735, 0.824] for social support).

### Differences in relevant outcome variables

Results from predicting outcome variables are displayed in Table 3. The *High Symptoms* profile (0.38) was significantly associated with a higher probability of endorsing suicidal ideation than the *High Numbing and Arousal* profile (0.16), which was also associated with a higher probability than the *Low Symptoms* profile (0.01). Only one significant difference emerged for attempted suicide, with those with a *High Numbing and Arousal* profile (0.02) endorsing a higher probability than those with a *Low Symptoms* profile (0.00). Participants with a *High Symptoms* profile (0.37) endorsed a significantly higher probability of seeking professional mental health care than those with a *High Numbing and Arousal* profile (0.17), who also reported a higher probability of seeking mental health care than those with a *Low Symptoms* profile (0.04).

**Table 3.** Comparisons for outcomes across profiles.

	Low Symptoms profile (85%)	High Numbing and Arousal profile (13%)	High Symptoms profile (2%)	
Outcomes	*Probability endorsing Yes compared to endorsing No*			Overall chi-square test
Suicidal ideation				
Yes	0.01	0.16	0.38	100.99***^a,b,c^
No	0.99	0.84	0.62	
Attempted suicide				
Yes	0.00	0.02	0.04	7.76*^a^
No	1.00	0.98	0.96	
Lifetime mental health service use				
Yes	0.04	0.17	0.37	73.32*** ^a,b,c^
No	0.96	0.83	0.63	

a = *Low symptoms profile* vs. *High numbing and arousal profile*, *p* < .001.

b = *Low symptoms profile* vs. *High symptoms profile*, *p* < .01.

c = *High numbing and arousal profile* vs. *High symptoms profile*, *p* < .001.

## Discussion

A three-profile model of PTSD symptoms, namely a *Low Symptoms* profile, *High Symptoms* profile, and *High Numbing and Arousal* profile, was found to be the best fitting in the current sample of Norwegian military veterans, which is in line with previous studies also finding three profiles (Armour et al., 2015; Contractor et al., 2015; Steenkamp et al., 2012). However, unlike previous studies on veterans and military personnel that have identified three latent profiles of PTSD and depression, generalized anxiety, and/or other comorbid psychopathology (Armour et al., 2015; Contractor et al., 2015; Jongedijk et al., 2019), this study investigated only latent profiles of PTSD symptoms, which may limit the comparability.

Given the low prevalence of probable PTSD (1.2%) in the sample, the small proportion of veterans within the *High Symptoms* profile (2%) was not surprising. Although our findings suggest that the profiles were primarily distinguishable by symptom severity (*Low Symptoms* profile and *High Symptoms* profile), a qualitative difference also emerged. One of the groups was characterized by specific symptom elevations (*High Numbing and Arousal)*, and the identification of this profile suggests that there is a noteworthy number of veterans who suffer from high numbing and arousal symptoms that would be unlikely to meet diagnostic criteria for PTSD. Compared to *Low* and *High symptoms* profiles, such symptom elevations have received less attention, even though there is evidence that intermediate profiles can result in distress comparable to those with clinically diagnosed PTSD (e.g., Marshall et al., 2001; Stein et al., 1997). In this study, we identified a subgroup of veterans who encounter difficulties specifically associated with increased levels of numbing and arousal symptoms, which has also been observed in previous studies (e.g., Hebenstreit et al., 2014; Maguen et al., 2013). These findings suggest that this group of individuals may possess distinct vulnerabilities and/or resilience factors that differentiate them from those who develop intrusion symptoms and a diagnosis of PTSD. This is supported by research on military service members that has found the presence of certain gene expressions to be attributed to the presence of intrusion symptoms (reexperiencing, nightmares, and flashbacks), whereas no gene expressions to be attributed to the other symptom clusters (Rusch et al., 2019). This raises the possibility that high numbing and arousal symptoms could indicate a separate stress-related disorder.

We also examined predictors of latent profile membership. In contrast to the results of a meta-analysis (Xue et al., 2015), this study discovered that a higher number of deployments was associated with a lower likelihood of belonging to the *High Symptoms* profile rather than the less symptomatic profile (i.e., *Low* or *High Numbing and Arousal*). One possible explanation for this finding is that the number of international deployments serves as a proxy of overall health. This suggests that military personnel who experience fewer mental or physical health problems are more likely to deploy frequently compared to those who experience more severe problems. Veteran women were more likely to belong to the *High Symptoms* profile compared to the two other profiles, or *High Numbing and Arousal* compared to *Low*, which is not unexpected, as research has shown that military women score higher on all PTSD symptoms than males (Hourani et al., 2015).

As expected, higher levels of social support were found to be significant predictors of belonging to the *Low Symptoms* profile. This is in line with previous studies showing that low social support is associated with PTSD symptoms (Wright et al., 2013; Xue et al., 2015; Zalta et al., 2021). Barriers to disclose traumatic experiences were associated with belonging to either the *High Numbing and Arousal* or *High Symptoms* profiles. Furthermore, barriers to disclose were associated with the *High Symptoms* profile as compared to the *High Numbing and Arousal* profile, but there were no significant differences between the two profiles regarding social support. This may indicate that veterans with *High Symptoms* and *High Numbing and Arousal* profiles perceive having similar levels of social support, but those with high overall symptoms may have more difficulties in utilizing this support (i.e., disclosing/sharing their experiences from Afghanistan).

Our findings are consistent with studies indicating that PMIEs are associated with symptoms of reliving/reexperiencing (Litz et al., 2018) and numbing symptoms (Presseau et al., 2019). Specifically, we found that individuals exposed to PMIEs were more likely to report symptoms of both reliving/reexperiencing and numbing symptoms (*High Symptoms* Profile), compared to the two other symptom profiles, or more likely to report higher numbing symptoms (*High Numbing and Arousal* profile) as compared to the *Low Symptoms* profile. Exposure to PMIEs was the only significant war zone trauma predictor in all multinomial logistic regressions, whereas experiences of personal threat and traumatic witnessing were not predictive of profile membership. This finding corroborates previous research indicating that PMIEs were among the war zone stressors that are most strongly associated with worse mental health outcomes (Presseau et al., 2019). Our finding suggests that PMIEs may have a unique and distinct impact on the development of PTSD symptoms in veterans compared to other types of war zone traumatic events, such as experiences of personal threat or traumatic witnessing, and veterans who experience PMIEs may be at a higher risk for severe PTSD symptoms. This finding also highlights the importance of considering PMIEs as a separate and distinct type of war zone trauma.

The results suggest that veterans who report high overall symptoms have a greater probability of endorsing suicidal ideation compared to those who belong in the *High Numbing and Arousal* or *Low Symptoms* profiles, which is in line with previous research indicating that suicide ideation is significantly and positively correlated with PTSD symptoms, and especially symptoms of altered cognition, mood, arousal, and reactivity (Brown et al., 2018). Furthermore, research has found that individuals with subclinical PTSD are at greater risk for suicide and suicidal ideation compared to individuals with no PTSD (Grubaugh et al., 2005; Marshall et al., 2001). This corresponds with our findings, which revealed that the intermediate PTSD profile characterized by high numbing/arousal was more likely to be associated with suicide attempts and suicidal ideation as compared to the *Low Symptom* profile. However, the *High Numbing and Arousal profile* was the only profile associated with a significantly higher probability of attempting suicide, as compared with the *Low Symptoms* profile. One explanation for this could be that individuals with *High Symptom* profiles may be more likely to have suicidal thoughts, but those with a *High Numbing and Arousal* profile may be more likely to act on those thoughts. Individuals with a *High Symptoms* profile may instead seek professional help, as demonstrated in our results. Such mental health help-seeking behavior might be protective, reducing the risk of suicide attempt among individuals in the high-risk group, while the individuals with an intermediate profile may, to a lesser degree, seek professional mental health care, and may not experience this buffering effect.

The implications of our study for the Norwegian Armed Forces and other countries are multifaceted. Our findings emphasize the significance of considering the heterogeneity in PTSD symptom presentation during the assessment, diagnosis, and treatment of veterans and military personnel. This underscores the need for a more nuanced and tailored approach to care, as veterans may fall into different symptom profiles, each requiring unique interventions. Specifically, veterans in the *High Numbing and Arousal* Profile, which is characterized by distinct symptom elevations, may not meet the standard diagnostic criteria for PTSD but still may need assistance and help from mental health care professionals. Instead, a more comprehensive understanding of symptom profiles is essential, which can lead to a more accurate estimation of the need for mental health services among veterans and military personnel.

The study needs to be interpreted in light of its strengths and limitations. The present study is the first to analyze the underlying structure of PTSD in a large sample of Norwegian military veterans. It is important to replicate studies across different countries, as cultural factors may affect the results and lead to different conclusions and interventions. However, there are also limitations to the study. First, the use of a self-report PTSD measure may affect the validity of participant symptom ratings. Second, the number of participants, and thus the statistical power, in certain groups (i.e., participants endorsing a suicide attempt in the *High Symptom* profile) might be too low to detect significant differences. Third, the study is cross-sectional and retrospective, which imply limitations with respect to causal inferences. For instance, the participants were asked about lifetime mental health service use and lifetime suicide attempts, which introduces ambiguity in associations with current PTSD symptom profiles. Of note, this study explored lifetime suicide attempt as an outcome variable, raising the possibility that the attempt occurred prior to the onset of PTSD symptoms. A suicide attempt is a life-threatening event, and as such, may be conceptualized as a DSM-5 Criterion A traumatic event (Stanley, Boffa, et al., 2019; Stanley, Hom, et al., 2019). Therefore, a suicide attempt may not solely be a consequence of PTSD but could also reflect an experience that culminates in PTSD (Stanley, Hom, et al., 2019). Fourth, the study did not test whether the latent variables differed as a function of other potential key variables, such as service status (still serving vs. left service) or method of administration of survey (online vs. mail). This may be relevant, as experiences and perspectives of those still serving may differ from those who have left service, and the method by which the individual was surveyed could also have affected profile membership. Taking the results and limitations into account, we encourage readers to use caution when comparing the results of the current study to those of other trauma-exposed populations or from other countries.

## Data Availability

Data are available from the corresponding author upon reasonable request.
